# Hemispheric Asymmetries in Radial Line Bisection: Role of Retinotopic and Spatiotopic Factors

**DOI:** 10.3389/fpsyg.2018.02200

**Published:** 2018-11-12

**Authors:** Sergio Chieffi, Giovanni Messina, Ines Villano, Antonietta Messina, Ciro Rosario Ilardi, Marcellino Monda, Monica Salerno, Francesco Sessa, Maria Pina Mollica, Gina Cavaliere, Giovanna Trinchese, Fabiano Cimmino, Paolo Murabito, Angela Catapano, Vincenzo Monda

**Affiliations:** ^1^Department of Experimental Medicine, Section of Human Physiology, Università degli Studi della Campania Luigi Vanvitelli, Naples, Italy; ^2^Department of Clinical and Experimental Medicine, University of Foggia, Foggia, Italy; ^3^Department of Experimental Medicine, Section of Human Physiology and Unit of Dietetics and Sports Medicine, Università degli Studi della Campania Luigi Vanvitelli, Naples, Italy; ^4^Department of Biology, University of Naples Federico II, Naples, Italy; ^5^Department of Clinical and Experimental Medicine, University of Catania, Catania, Italy

**Keywords:** line bisection, radial lines, distal bias, spatiotopic factors, retinotopic factors, hemispheric asymmetry

## Abstract

Previous studies showed that healthy individuals bisect radial lines oriented along the midsagittal plane farther than the true center (distal bisection bias). It was proposed that the distal bisection bias depended on the presence of an attention bias directed toward far space (distal attention bias) and that this bias is related to the activity of the occipitotemporal visual processing stream. Other studies have also suggested that a similar distal attention bias is linked to the activity of the right hemisphere. In the present experiment we investigated whether distal bisection bias increased when radial lines were placed in the left hemispace. Furthermore, we also examined whether the bisection bias was enhanced by the use of the left hand, as left hand movements are mainly controlled by the right hemisphere. Right-handed participants were asked to bisect radial lines presented below eye level along the midsagittal plane (central lines), or laterally and parallel to the midsagittal plane, in the left or right hemispace (left and right lines, respectively). Participants used their right or left hand. The results showed that participants consistently bisected left and central radial lines farther than (i) the true center and (ii) the subjective midpoint of right radial lines. Conversely, they bisected accurately right radial lines. The hand did not influence bisection error. The present study suggests that the distal bisection bias found in the bisection of left radial lines might depend on the presence of a distal attention bias related to right hemisphere activity. The relative contribution of retinotopic and spatiotopic factors in producing the distal bisection bias is discussed.

## Introduction

Line bisection is a perceptual-motor task in which participants are asked to localize and mark with a pencil the center of a line drawn on a sheet of paper. The task is commonly used in neurological examinations for assessing hemispatial neglect. When patients with hemispatial neglect bisect horizontal lines, they place the subjective midpoint toward the ipsilesional side (Bisiach and Vallar, 1988). Neglect can also occur along radial and vertical dimensions. Occipitoparietal damage may produce near/lower space neglect (Rapcsak et al., 1988; Butter et al., 1989; Mennemeier et al., 1992), occipitotemporal damage far/upper space neglect (Shelton et al., 1990; Adair et al., 1995). In the first case, patients bisect radial and vertical lines, respectively, farther and more above the true center; in the second case, nearer and more below the true center. Drain and Reuter-Lorenz (1996) hypothesized that the occipitotemporal (ventral) stream shifts attention toward far/upper space, the occipitoparietal (dorsal) stream toward the near/lower space. Furthermore, they suggested that the two streams are in mutually inhibitory control of attention orienting. Damage to occipitoparietal (occipitotemporal) regions would lead to a concomitant disinhibition in occipitotemporal (occipitoparietal) activity and a far/upward (near/downward) orienting bias (Drain and Reuter-Lorenz, 1996).

Bisection performance has also been studied in neurologically healthy individuals. Note that the errors of healthy individuals are much smaller than those made by neglect patients. Different factors may influence bisection performance, such as spatial orientation (Bowers and Heilman, 1980; Shelton et al., 1990; Chieffi, 1996), learned reading direction (Chokron and Imbert, 1993), presence of contextual stimuli (Toba et al., 2011; Chieffi et al., 2012, 2014a; Chieffi, 2016), and the age of the subjects (Chieffi et al., 2014b). Regarding the spatial orientation of the line to be bisected, some researchers reported a systematic leftward bias in bisection of horizontal lines (Bowers and Heilman, 1980). This phenomenon was called pseudoneglect. Conversely, other authors found consistent rightward errors (Halligan and Marshall, 1989) or failed to find any constant error (Halligan et al., 1991). The bisection bias of healthy individuals for lines oriented along the radial or vertical axis appears more consistent, with radial lines bisected farther than the true midpoint and vertical lines bisected above the true midpoint (Shelton et al., 1990; Chieffi, 1996, 1999). A possible explanation for the distal bisection bias observed in radial line bisection is that the attentional bias toward far space (ventral stream) prevailed over the attentional bias toward near space (dorsal stream). Assuming that participants foveate the central region of the line (Ishiai et al., 1989) to localize the subjective midpoint, the image of the distal portion of the line is projected onto the inferior retina (and processed primarily by the ventral stream), the image of the proximal portion onto the superior retina (and processed primarily by the dorsal stream). This might have magnified the magnitude of the distal portion of the line. Previous studies showed that the magnitude of attended stimuli appears magnified compared to that of unattended stimuli (Fraisse et al., 1956; Milner et al., 1992; Prinzmetal and Wilson, 1997; Masin, 2003). Such a magnification of the distal portion of the line might have moved forward the location of the subjective midpoint.

An important question is whether attentional influence acts within a retinotopic or spatiotopic frame of reference. In the first case, the projection of the line onto the retina would be determinant (Previc, 1998); in the second case, the position of the line in space with respect to the participant’s body (Shelton et al., 1990; Chieffi et al., 2008). Geldmacher and Heilman (1994) suggested that both retinotopic and spatiotopic factors may influence radial line bisection. In their experiment, the authors asked participants to bisect radial lines presented either below or above eye level. When the lines were placed below eye level, the direction of retinotopic and spatiotopic effects coincided. Conversely, when the radial lines were positioned above the eyes, the direction of retinotopic and spatiotopic effects was in conflict, being the distal portion of the line projected onto the superior retina (dorsal stream). In below condition participants bisected radial lines farther than the true center. Conversely, bisection errors did not differ from zero in above condition (Geldmacher and Heilman, 1994). In other words, in the latter condition, retinotopic and spatiotopic effects seemed to balance one another. However, other factors might contribute to the distal bias, such as asymmetric eye scanning from far to near (Halligan and Marshall, 1993), or the “magnification” of far objects to compensate for a known reduction in size as a function of stimulus distance from the subject (Barrett et al., 2002).

Experimental evidence suggests that not only the ventral stream, but also the right hemisphere may shift attention away from the body (Heilman et al., 1995; Roth et al., 2002; Szpak et al., 2016). Heilman et al. (1995) asked participants to compare the size of two radial lines presented, at eye level, one in the left and the other in the right hemispace. The authors found that the lines presented in the left hemispace appeared shorter than those presented in the right hemispace. Heilman et al. (1995) suggested that two factors could have played a determining role: the retinal projection of the line and the differences between the two hemispheres in orienting attention. Regarding the retinal projection of radial lines, note that the proximal portion of left lines was projected on the right half of the retina of both eyes (and the information processed by the right hemisphere); the distal portion was projected on the left half of the retina of both eyes (and processed by the left hemisphere). The opposite occurred when the lines were placed to the right. Regarding the differences between the two hemispheres in orienting attention, Heilman et al. (1995) suggested that the left hemisphere directed attention toward the body, the right hemisphere away from body. Accordingly, when the radial lines were placed on the right, attention was directed toward the ends of the lines; when the lines were placed on the left, attention was directed toward the middle of the lines, producing an underestimation of their length. Subsequently, Roth et al. (2002) studied monocular bisection of radial lines. They asked right and left eye dominant participants to bisect radial lines located in either the left or right hemispace, using one eye (the other eye was patched). Monocular eye patching is thought to produce preferential activation of attentional systems contralateral to the viewing eye (Posner and Rafal, 1987). Roth et al. (2002) found that right eye dominant subjects showed a distal bisection bias when they used the left (non-dominant) eye and bisected left radial lines. The authors suggested that in this experimental condition the right hemisphere was preferentially activated as it received fibers coming from the nasal retina. Nasal fibers are more numerous, have a higher density, greater diameter, and transmit information faster than fibers coming from the temporal retina and projecting to the left hemisphere (Bishop et al., 1953; Perry et al., 1984; Curcio et al., 1987). More recently, Szpak et al. (2016) examined hemispheric differences in spatial orienting of attention along the radial dimension by employing a landmark line bisection task. The landmark task is a perceptual task in which participants have to judge whether a transection mark appears closer to one or the other end of the line. Szpak et al. (2016) found that the distal bias was stronger for lines presented in the left hemispace than in the right hemispace.

In the current experiment, we examined whether the attentional bias toward far space, presumably linked to right hemisphere activity, influenced the location of the subjective midpoint in a radial line bisection task. We examined also whether the hand used influenced the localization of the subjective midpoint. Since hand movements are mainly controlled by the contralateral hemisphere (Levine et al., 1978; Fisk and Goodale, 1988), they might enhance contralateral hemisphere activation and influence bisection performance. The participants bisected radial lines presented either along the midsagittal plane or laterally and parallel to the midsagittal plane, in the left or right hemispace. Bisection was performed using the right or left hand. Our predictions were as follows. According to previous observations (Roth et al., 2002; Szpak et al., 2016), participants localize the subjective midpoint of lines presented in the left hemispace farther than that of lines presented in the right hemispace. If this was true, the use of the left hand might enhance the distal bias observed in the bisection of the left lines.

## Materials and Methods

### Participants

Twenty-eight healthy, right-handed subjects (17 women and 11 men) participated in the study. Their mean age was 24.1 years (SD 4.2, range 19 – 34). The Edinburgh Handedness Inventory (Oldfield, 1971) was used to measure handedness (mean score = 95.3, *SD* = 4.72, range = 85–100). All the subjects reported having normal or corrected-to-normal vision. The experiment was approved by the ethics committee and was performed in accordance with the 1964 Declaration of Helsinki. Participants gave written informed consent to take part in the study.

### Stimuli

The stimuli were black lines 24 cm long and 2.0 mm wide. They were drawn and centered on a sheet of white paper 29.7 cm × 21.0 cm.

### Procedure

**FIGURE 1:**
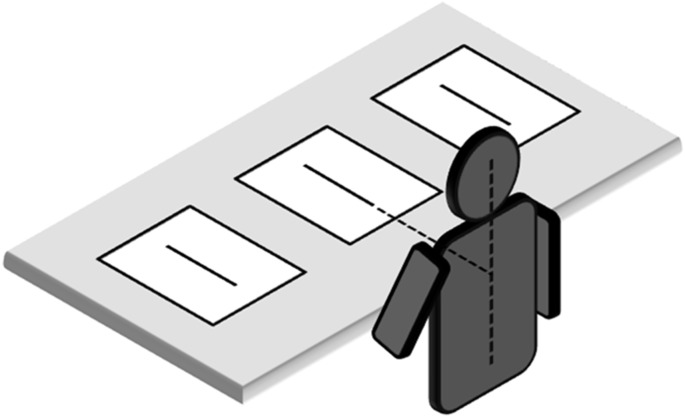
Placement of line bisection stimuli in relation to subject. Note that proportions of elements are not naturalistic.

The participants sat in a comfortable chair in front of a table. The table was 75 cm high. The chair height was adjustable in order to ensure that the vertical distance between the table surface and the eyes was about 45 cm. Stimuli were presented radially on the table top (Figure 1). They could be located at three different spatial locations: (i) along the midsagittal plane (line midpoint: 40 cm from the subject’s trunk) or laterally (ii) 30.5 cm to the right or (iii) 30.5 cm to the left of the midsagittal plane. Hand starting position was along the midsagittal axis, about 10 cm from the trunk. The participants were asked to bisect the lines with a pencil, using their right or left hand. They were allowed to freely move the eyes and head, but not their trunk. Trunk vertical axis served as a reference for left and right positions in space. Each participant bisected a total of 36 lines, administered in 6 blocks ((right hand vs. left hand) x (left hemispace vs. central position vs. right hemispace)) of trials. The sequence in which each hemispace and hand were tested was randomized in one half of the participants. In the other half, the reverse sequence was used. Line bisection error (LBE) was measured as the distance of the subjective midpoint from the true center. Errors farther than the true center were assigned a positive value, and errors nearer than the true center were given negative values. The standard deviation of the LBE calculated for each condition was the variability of bisection error (VBE). Variability scores quantify the scatter of subjective midpoints and are sensitive to variability or inconsistency in responding.

### Statistical Analysis

The mean values of LBE and VBE were analyzed. They were subjected to two-way analyses of variance with hand (right vs. left) and spatial location (left hemispace vs. central position vs. right hemispace) as the within-subjects factors. Paired comparisons were performed using Bonferroni procedure. Furthermore, to investigate the direction of misbisection in each experimental condition, one-sample, two-tailed *t* tests (df = 27) were also performed comparing LBE with the null set (true center). Significance level was fixed at *p* = 0.008, considering an overall 0.05 level divided by the number of comparisons, according to Bonferroni procedure.

## Results

**FIGURE 2:**
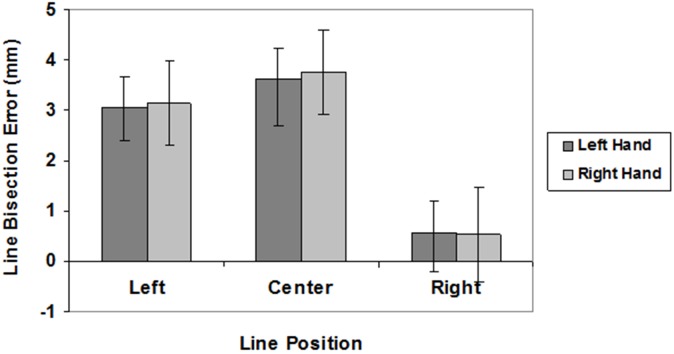
Line bisection errors in different line position conditions (left, center, right) for the left (dark gray) and right (light gray) hand. Mean values are shown with SE (bars).

**FIGURE 3:**
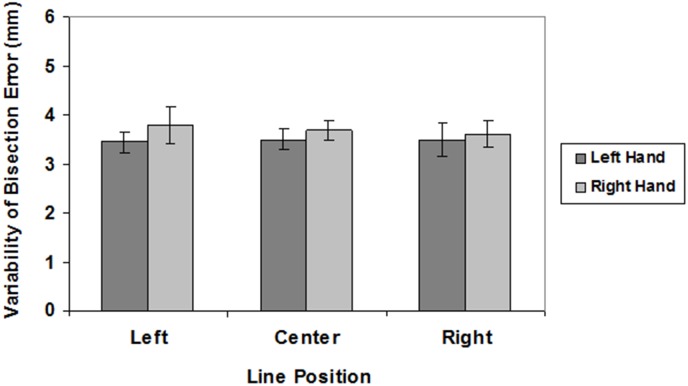
Variability of bisection errors in different line position conditions (left, center, right) for the left (dark gray) and right (light gray) hand. Mean values are shown with SE (bars).

The mean values of LBE and VBE are graphically reported in Figures 2, 3, respectively.

Analyses of LBE scores showed a significant main effect of spatial location (*F*(2,54) = 11.01, *P* < 0.0001; left hemispace = 3.09 mm; central position = 3.69 mm; right hemispace = 0.54 mm). *Post hoc* analyses revealed that subjects bisected radial lines placed in the left hemispace and in central position farther than radial lines placed in the right hemispace (*p* < 0.005). There was no significant effect of hand (*F*(1,27) < 1; right hand = 2.47 mm, left hand = 2.41 mm), and no interaction (F(2,54 ) < 1). One-sample, two-tailed *t*-tests (df = 27) showed that subjects bisected central and left radial lines farther than the true center both with the right and left hand (*p* < 0.001).

There were no significant main effects on VBE (hand: *F*(1,27) = 2.01, n.s.; spatial location: *F*(2,54) < 1) and no interaction (*F*(2,54 ) < 1).

## Discussion

The main findings of the present experiment were: (i) subjective midpoints were located farther away for left and central radial lines than for right radial lines and the true center; (ii) contrary to what was predicted, the hand did not influence the bisection error.

It is worth emphasizing that in our study the lines were placed below eye level and the participants were free to move their eyes and head. For this reason, there might have been significant differences in the way in which the images of the central and lateral lines were projected onto the retina. As will be described in more detail below, while the image of central lines was projected to the inferior and superior retina, the image of lateral lines was projected on opposite retinal quadrants. We therefore will examine separately participants’ bisection performance in the central and lateral conditions.

**FIGURE 4:**
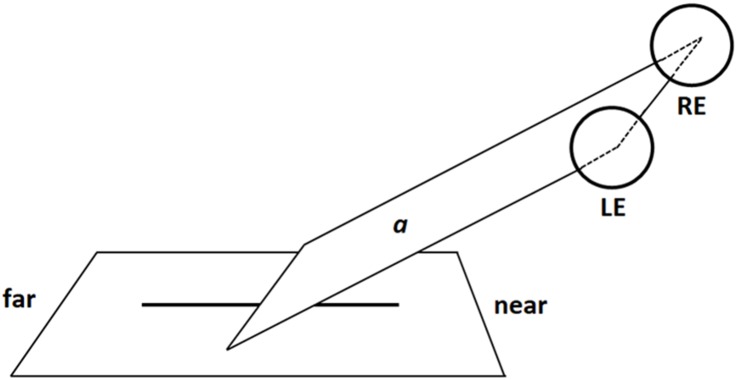
Schematic illustration of a virtual gaze plane (a) crossing the radial line in central viewing condition (LE: Left Eye; RE: Right Eye). Note that proportions of elements are not naturalistic.

**FIGURE 5:**
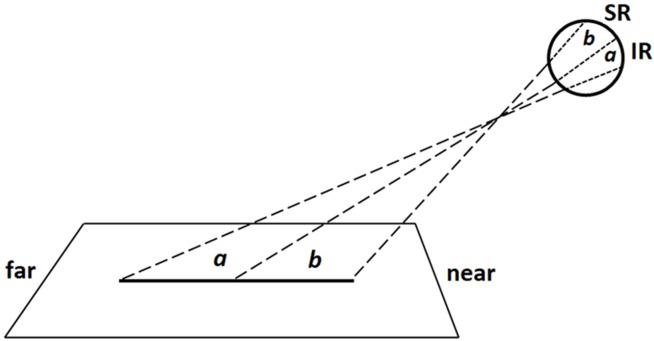
Schematic illustration of retinal projections in central viewing condition: the image of distal line portion (a) is projected onto the inferior retina (IR), whereas the image of proximal portion (b) is projected onto the superior retina (SR). Note that proportions of elements are not naturalistic.

To understand how the images of radial lines were projected onto the retina, imagine a virtual plane passing through the participants’ gaze and crossing the line to be bisected (Figure 4). When the radial line was located along the midsagittal plane, the gaze plane crossed the line perpendicularly so that (i) the image of the distal portion was projected onto the inferior retina (and the information processed primarily by the ventral stream); (ii) the image of the proximal portion was projected onto the superior retina (and the information processed primarily by the dorsal stream) (Figure 5). In line with previous research (Shelton et al., 1990; Chieffi, 1996, 1999), we found that participants consistently bisected central radial lines farther than the true center. As mentioned in the introduction, both retinotopic and spatiotopic factors might have contributed to the distal bias.

**FIGURE 6:**
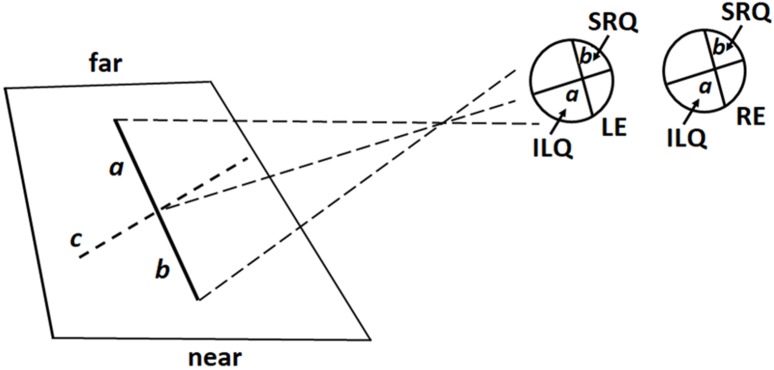
Schematic illustration of retinal projections in lateral (left) viewing condition: the virtual gaze plane crosses the line diagonally (c) so that the image of its distal portion (a) is projected onto the inferior left retina quadrant (ILQ), whereas the image of its proximal portion (b) is projected onto the superior right retina quadrant (SRQ) of both eyes. LE, Left Eye; RE, Right Eye. Note that proportions of elements are not naturalistic.

When the radial line to be bisected was located laterally to the midsagittal plane, in either the left or right hemispace, participant’s gaze plane crossed the line to be bisected diagonally (Figure 6). In this way, the images of the distal and proximal portions of the line were projected onto opposite retinal quadrants. More precisely: (1) for left radial lines, the image of the distal portion was projected onto the inferior left retinal quadrants (and the information processed by the left ventral stream); the image of the proximal portion onto the superior right retinal quadrants (and the information processed by the right dorsal stream) (Figure 6); (2) for right radial lines, the image of the distal portion was projected onto the inferior right retinal quadrants (and the information processed by the right ventral stream); the image of the proximal portion was projected onto the superior left retinal quadrants (and the information processed by the left dorsal stream). Participants consistently bisected left radial lines farther than (i) the subjective midpoint of right radial lines and (ii) the true center. In contrast, bisection errors did not differ from zero error when the lines were presented in the right hemispace. These findings are in line with previous observations (Roth et al., 2002) showing that right eye dominant subjects display a distal bias when they used the left eye and bisected left radial lines. The authors (Roth et al., 2002) proposed that (i) hemispheric attentional systems contralateral to the viewing eye were relatively activated compared with those contralateral to the patched eye and (ii) the right hemisphere, in right eye dominant subjects, was biased toward far space. From retinal viewpoint, in both our and Roth et al.’ (2002) studies, the information about the distal portion of the left line was processed by the left ventral stream. Since the ventral stream is suggested to shift attention toward far space, retinotopic factors linked to the processing of visual information by the left ventral stream might have produced the distal bias. We hold this to be a weak hypothesis because also the visual information coming from the distal portion of the right radial line was processed by the ventral stream, more precisely the right ventral stream. Thus, if the distal bias observed in the bisection of left radial lines depended on retinotopic factors, we should hypothesize that the the attentional bias toward far space was greater in the left than the right ventral stream. A more plausible hypothesis is that spatiotopic factors, related to the position of the line in space, in a body-centered coordinate system, played an important role in modulating the distal bias observed in the bisection of the left radial lines. This hypothesis is based on and supported by experimental observations indicating that: (i) activities in the left hemispace are related to right hemispheric mechanisms (Kinsbourne, 1972; Coslett et al., 1993; Coslett, 1999) and (ii) the right hemisphere is involved in shifting attention toward far space (Heilman et al., 1995; Weiss et al., 2000; Roth et al., 2002; Szpak et al., 2015, 2016).

With respect to the first point, studies performed on healthy and brain-damaged individuals suggest that stimuli presented in one hemispace, defined on the basis of a body-centered coordinate system, preferentially engage contralateral hemisphere systems. Kinsbourne (1972) showed a close relationship between leftward eye and head movements and right hemisphere activation, and between rightward eye and head movements and activation of the left hemisphere. Furthermore, Coslett and colleagues (Coslett et al., 1993; Coslett, 1999) found that brain-damaged subjects with either left or right hemispheric damage performed more poorly when stimuli were presented in the contralesional hemispace. Conversely, when stimuli were shifted in the ipsilesional, “good” hemispace, by manipulating head or body position, performance improved (Coslett et al., 1993; Coslett, 1999).

Experimental evidence suggests that the right hemisphere is involved in shifting attention toward far space. As previously reported, this hypothesis was first proposed by Heilman et al. (1995) and received subsequent support by other researchers (Weiss et al., 2000; Roth et al., 2002; Szpak et al., 2015, 2016). Weiss et al. (2000) recorded brain activity using position emission tomography while participants bisected horizontal lines and pointed to dots in near and far space. When participants performed either task in near space there was increased activation of areas in the left hemisphere such as the dorsal occipital cortex, intraparietal cortex, ventral premotor cortex. Conversely, when participants carried out either task in far space there was bilateral activation of the ventral occipital cortex and activation of the right medial temporal cortex. Szpak et al. (2015) asked participants to make closer/further judgments about the relative location of two 3D spheres located in the left vs. right hemispace. Results demonstrated that participants judged the sphere located on the left to be farther than the sphere on the right.

Regarding the variability of bisection error, there was no effect of the hand used or the position of the line. Variability scores quantify the scatter of subjective midpoints and are sensitive to variability or inconsistency in responding. Therefore, in our experiment, the consistency of bisection performance was similar in all the conditions examined.

It is generally recognized that the activities of each hand are programmed and controlled mainly by the contralateral hemisphere (Levine et al., 1978; Fisk and Goodale, 1988). Therefore, we expected that the use of the left hand, enhancing right hemisphere activation, might produce an increase of the distal bisection bias in the left line condition. Contrary to what was predicted, we did not observe any influence by the hand used on bisection error. Interestingly, in functional (f)MRI studies of unilateral hand motor performance, although some researchers found strictly contralateral cortical motor activation (Catalan et al., 1998; Bütefisch et al., 2005), other investigators observed bilateral activation (Winstein et al., 1997; Seidler et al., 2004; Diedrichsen et al., 2013). Thus, it is possible that the absence of a hand effect on radial line bisection depended on bilateral hemispheric activation related to hand bisection movement.

On the whole, the results of the present study suggest the existence of two attentional systems involved in shifting attention toward far space. A first system is represented by the ventral visual processing stream. Ventral system not only receives and processes information from far space, but it also shifts attention toward far space. In this way, the distal portion of the central radial line was magnified and participants mark the subjective midpoint of the line farther than the true center. Furthermore, both retinotopic (retinal projection of line) and spatiotopic (line position/distance from participant’s body) factors appear to contribute to the distal bias. A second system involved in shifting attention toward far space is represented by the right hemisphere. This hypothesis was supported by the observation that participants consistently bisected the radial lines presented in the left hemispace farther than the true center. Plausibly, the localization of the line in the left hemispace (spatiotopic factors) produced an activation of the right hemisphere that, in turn, displaced attention toward far space, magnifying in this way the distal portion of the line and thereby causing a distal bias in line bisection.

## Author Contributions

SC, GM, IV, AM, CI, and VM conceived the study and participated in its design. SC, GM, MM, MS, FS, MPM, and VM contributed to the conception and design. SC, GM, MM, GC, GT, FC, AC, and VM wrote the manuscript. SC, GM, IV, AM, CI, MS, FS, and VM drafted the article and revised it critically for important intellectual content. SC, GM, MPM, GC, GT, FC, PM, AC, and VM provided the final approval of the version to be published. All authors read and approved the final manuscript.

## Conflict of Interest Statement

The authors declare that the research was conducted in the absence of any commercial or financial relationships that could be construed as a potential conflict of interest.
